# Left Ventricular Systolic Function Has Strong Independent Genetic Background from Diastolic Function: A Classical Twin Study

**DOI:** 10.3390/medicina57090935

**Published:** 2021-09-05

**Authors:** Andrea Ágnes Molnár, Márton Kolossváry, Bálint Lakatos, Márton Tokodi, Ádám Domonkos Tárnoki, Dávid László Tárnoki, Attila Kovács, Bálint Szilveszter, Szilard Voros, György Jermendy, Pál Maurovich-Horvat, Béla Merkely

**Affiliations:** 1 Heart and Vascular Center, Semmelweis University, 1122 Budapest, Hungary; martonandko@gmail.com (M.K.); lakatosbalintka@gmail.com (B.L.); tokmarton@gmail.com (M.T.); kovatti@gmail.com (A.K.); szilveszter.balint@gmail.com (B.S.); maurovich.horvat@gmail.com (P.M.-H.); merkely.bela@gmail.com (B.M.); 2 Medical Imaging Center, Semmelweis University, 1082 Budapest, Hungary; tarnoki2@gmail.com (Á.D.T.); tarnoki4@gmail.com (D.L.T.); 3 Scientific Affairs, Global Institute for Research, LLC, Richmond, VA 23219, USA; szilard.voros@globalgenomicsgroup.com; 4 3rd Department of Internal Medicine, Bajcsy Zsilinszky Hospital, 1106 Budapest, Hungary; gyjermendy@gmail.com

**Keywords:** left ventricle, speckle tracking, twin, genetic, environmental, co-heritability

## Abstract

*Background and Objectives:* No data are available on whether the heritability of left ventricle (LV) systolic and diastolic parameters are independent of each other. Therefore, our aim was to assess the magnitude of common and independent genetic and environmental factors defining LV systolic and diastolic function. *Materials and Methods:* We analyzed 184 asymptomatic twins (65% female, mean age: 56 ± 9 years). Transthoracic echocardiography was performed to measure LV systolic (global longitudinal and circumferential strain; basal and apical rotation) and diastolic (early diastolic velocity of mitral inflow and lateral mitral annulus tissue; deceleration time and early diastolic strain rate) parameters using conventional and speckle-tracking echocardiography. Genetic structural equation models were evaluated to quantify the proportion of common and specific genetic (Ac, As) and environmental factors (Ec, Es) contributing to the phenotypes. *Results:* LV systolic parameters had no common genetic or environmental heritability (Ac range: 0–0%; Ec range: 0–0%; As range: 57–77%; Es range: 24–43%). Diastolic LV parameters were mainly determined by common genetic and environmental effects (Ac range: 9–40%; Ec range: 11–49%; As range: 0–29%; Es range: 0–51%). Systolic parameters had no common genetic or environmental factors (Ac = 0%; Ec = 0%) with diastolic metrics. *Conclusions:* Systolic LV parameters have a strong genetic predisposition to any impact. They share no common genetic or environmental factors with each other or with diastolic parameters, indicating that they may deteriorate specifically to given effects. However, diastolic functional parameters are mainly affected by common environmental influences, suggesting that pathological conditions may deteriorate them equally. Estimation of the genetic and environmental influence and interdependence on systolic and diastolic LV function may help the understanding of the pathomechanism of different heart failure classification types.

## 1. Introduction

Heart failure (HF) is a highly prevalent, multi-factorial progressive disease associated with substantial morbidity and mortality, thus representing one of the main global health problems [1]. The etiology of the disease can be based on genetic or acquired factors that can affect left ventricular (LV) systolic and diastolic function [1]. Transthoracic echocardiography is the standard diagnostic method to assess LV systolic and diastolic function by measuring conventional LV ejection fraction (LVEF), mitral inflow and mitral annular tissue doppler velocities. LVEF assessment is crucial in HF classification; thus, we can differentiate HF with reduced EF (HFrEF), moderately reduced EF (HFmrEF) or preserved EF (HFpEF). Diastolic dysfunction is the main mechanism of HFpEF, representing up to 50% of patients with HF and resulting in similar mortality rates to those of HFrEF. However, our knowledge of HFpEF is still limited regarding pathophysiology, clinical treatment, and predisposing and precipitating factors [1]. Advanced imaging techniques such as strain analysis using speckle-tracking echocardiography have higher sensitivity to detect subclinical stages of different cardiac alterations and overall represent a more accurate LV functional assessment compared with conventional EF measurement [2,3]. Strain analysis describes the myocardial deformation—the extent to which the LV shortens along the long axis (global longitudinal strain (GLS)), the LV cavity size decreases in circumference (global circumferential strain (GCS)), and the LV wall thickens (global radial strain (GRS)). Furthermore, the commonly used early diastolic strain rate (LsrE) represents LV enlargement rate during early diastole [4].

Classical twin studies use genetic structural equation models to quantify the proportion of genetic and environmental factors contributing to the given phenotype. This model is based on the fact that monozygotic (MZ) twins share 100% of their genes, while dizygotic (DZ) twins share only half. It is possible to decompose the variation of a measured parameter between the twin siblings into additive genetic factors (A), common environmental factors (C, which are similar between twins), and unique environmental factors (E, which are specific to one of the siblings). Additive genetic influence (A) describes the additive effect of multiple genes, while non-additive genetic factors (NA) represent interactive effects of different alleles and include genetic dominance within locus interaction (D). Shared environmental effects (C) and non-additive genetic effects are confounded (D), and the choice between them is based on the pattern of twin correlations. Previously, we have demonstrated that mathematical modelling of genetic influence shows high systolic myocardial deformation heritability in a Caucasian population without systolic dysfunction. However, environmental factors have a stronger influence on LV diastolic function [5]. Furthermore, comparably higher LV systolic functional heritability was found in Asian and American population-based family studies as well [6,7,8]. Systolic and diastolic functional alterations may develop independently in different heart failure phenotypes. To date, no data are available on whether the heritability of LV systolic and diastolic function is independent of each other or if there is a common genetic or environmental influence that drives pathological conditions.

Therefore, the aim of our study was to assess whether there are common genetic or environmental factors that determine systolic functional parameters, diastolic functional parameters or both. Using accurate LV functional measurements and appropriate statistical methods might give a more precise estimation of the influence and interdependence of different genetic and environmental predisposing factors on systolic and diastolic LV function.

## 2. Materials and Methods

### 2.1. Study Population

The investigation was part of the BUDAPEST-GLOBAL (Burden of Atherosclerotic Plaques Study in Twins—Genetic Loci and the Burden of Atherosclerotic Lesions) clinical study, which is a prospective, single-center, classical twin cohort study established to assess the effects of genetic and environmental factors on different cardiovascular phenotypes [9]. The twin participants were recruited from the Hungarian Twin Registry [10]. Detailed enrollment and exclusion criteria were previously published. In brief, twin pairs with obstructive coronary artery disease (indicated by coronary CT angiography), any cardiomyopathy, severe valvular heart disease or symptoms of heart failure were excluded [5,9]. All twin siblings provided written consent for participation. The study design fulfilled the terms of the Declaration of Helsinki and the protocol was approved by the Scientific and Research Ethics Committee of the National Medical Research Council (Approval number: 58401/2012/EKU (828/PI/12), amendment-1: 12292/2013/EKU (165/2013)).

Basic anthropometric parameters (weight, height, waist circumference) of every person were measured. Past medical history and lifestyle habits were recorded. The physical examination included a blood pressure measurement, a 12-lead ECG, and an assessment of potential symptoms and physical signs of any cardiovascular disease.

### 2.2. Echocardiography Measurements

Transthoracic echocardiography (iE33 system, S5-1 transducer, Philips Healthcare, Best, The Netherlands) was performed. LV end-diastolic and end-systolic volumes and ejection fraction were calculated using the biplane Simpson method. The Devereux formula was used to quantify LV mass. Parameters of LV diastolic function (i.e., mitral inflow velocities, pulsed-wave tissue Doppler imaging of mitral medial and lateral annular velocities) were determined according to current guidelines. Mitral inflow parameters included early diastolic inflow velocity (E wave), late diastolic inflow velocity (A wave), and deceleration time of E wave (DCT), even though tissue doppler examination measures lateral mitral annulus early diastolic tissue velocity (EeLat). Body surface area (BSA) was calculated by the Mosteller formula [11].

Beyond conventional echocardiographic measurements, the protocol included the acquisition of high-quality images appropriate for two-dimensional speckle-tracking analysis. Parasternal short-axis views of the mitral valve, papillary muscle, and apical levels, as well as apical four-chamber, two-chamber, and long-axis views were acquired for speckle tracking. Raw data were exported to a standalone workstation for off-line analysis with commercially available software (2D Cardiac Performance Analysis v1.2, TomTec Imaging Systems, Unterschleissheim, Germany). After manual delineation of the endocardial surface, the software tracked the region of interest throughout three consecutive cardiac cycles. Peak systolic strains of the 16 LV segments averaged over three cardiac cycles were used to calculate the corresponding global values. GCS and GRS were quantified using the parasternal short-axis views. In addition, we determined the apical counterclockwise and basal clockwise rotations by averaging the four and six segmental values, respectively. Apical views were used to assess GLS and systolic strain rate, along with early diastolic strain rate values. Subjects with fewer than ten available segmental values were not included in the statistical analysis.

### 2.3. Statistics

Genetic structural equation models were evaluated to quantify the proportion of genetic and environmental factors contributing to the given phenotype. Based on the fact that MZ twins share 100% of their genes, while DZ twins only share half, it is possible to decompose the variation between the twin siblings into additive genetic factors (As), common environmental factors (Cs) that are similar between the twins and unique environmental factors (Es) that are specific to one of the siblings [12]. Submodels of the full ACE models were calculated to determine the most parsimonious model capable of correctly describing our data. If a submodel did not consider all three factors to be present and only AC, CE, or E was found to have a non-significant deterioration in fit as compared with the full ACE model using the likelihood test, the more parsimonious model with the better fit was selected. Last, we fit independent pathway models to our data. Using independent pathway models, it is possible to further partition the sources of variation between the phenotypes into common additive genetic factors (Ac), which refers to genes that determine all the investigated phenotypes; common environmental factors (Cc), which are factors that drive the heritability of the investigated traits together; and common unique environmental factors (Ec), which are environmental factors that are specific to one of the siblings but affect all measured parameters in the model. The remaining variation is partitioned into As, Cs, and Es factors specific to the given trait, which is not shared with other traits. Therefore, the total heritability of a given phenotype is the sum of the common and the specific factors. Submodels investigating the possibility of excluding one of these sources of variation for each trait were calculated. The most parsimonious submodel that did not show significant deterioration in fit using the likelihood test as compared with the full model was selected.

Three statistical models were considered. A Systolic Model, which assess the heritability and co-heritability of GLS, GCS, basal rotation, and apical rotation; a Diastolic Model using LSrE, EeLat, and DCT; and a Systolo-Diastolic Model considering GLS, Lsre, EeLat, and DCT were calculated. All calculations were adjusted for age and sex. All analyses were performed using R version 3.4.0 [13]. Twin modelling was performed using OpenMx version 2.7.18 [14]. A p value lower than 0.05 was considered significant.

## 3. Results

In the current study, we included 184 twin subjects (92 twin pairs, 65% female, 54 MZ, and 38 same-gender DZ twin pairs). We excluded nine twin pairs from the original BUDAPEST-GLOBAL cohort because of inadequate echocardiographic image quality [5,9].

### 3.1. Patient Demographics

Demographic characteristics and basic echocardiographic parameters of the study population were published previously by our workgroup [5]. Briefly, our twin cohort represented a middle-aged Caucasian population (age: 56 ± 9 years), with a representative prevalence of hypertension (40%, 74/184 siblings), diabetes mellitus (8%, 15/184 siblings), and dyslipidemia (41%, 76/184 siblings) to the general population. DZ twin pairs were significantly older and had a higher heart rate.

### 3.2. Echocardiography Results

LV systolic function, measured by both ejection fraction and GLS, was normal in every sibling (EF: 59 ± 7%, GLS: −22.0 ± 2.6%). Early diastolic mitral inflow velocity (E wave) was higher in MZ twin pairs (E wave: 0.74 ± 0.16 m/s) compared with the DZ twin pairs (E wave: 0.70 ± 0.12 m/s). All other and echocardiographic parameters, including strain and strain rate measurements, were in normal range and comparable between the MZ and DZ pairs. Detailed comparisons were published earlier [5].

### 3.3. Genetic Structural Equations

When considering the Systolic Model on the common and specific genetic and environmental factors contributing to LV myocardial deformation, interestingly, we found strong genetic background of strain parameters. However, there were no common genetic or environmental determinations among GLS, GCS, and basal and apical rotation (Table 1).

The specific genetic contribution to the variance of strain parameters ranged between 61% and 75%, while the common genetic or environmental contribution accounted for 0%. We found some specific environmental effects for each LV systolic strain parameter, with relatively higher contribution to the variance in the case of basal and apical rotation (43% vs. 36%). GLS and GCS were only slightly affected by specific environment (23% vs. 24%).

On the contrary, the investigated diastolic parameters in the Diastolic Model (LSrE, EeLat, and DCT) were rather determined by environmental effects (Table 2). The overall environmental contribution to the variance to diastolic function ranged between 49% and 62%, both by common and by specific factors. Specific environmental effects were responsible only for LSrE and DCT variance in 37% and 51%, respectively. Nevertheless, all diastolic parameters shared common environmental influence. Moreover, common genetic determination of LSrE, EeLat, and DCT diastolic traits could also be determined in 40%, 25%, and 9%, respectively. Despite higher genetic determination of LSrE (40%), we found no specific component of genetic influence on this diastolic trait; only EeLat and DCT showed 26% and 29% specific inheritance, respectively.

**Figure 1:**
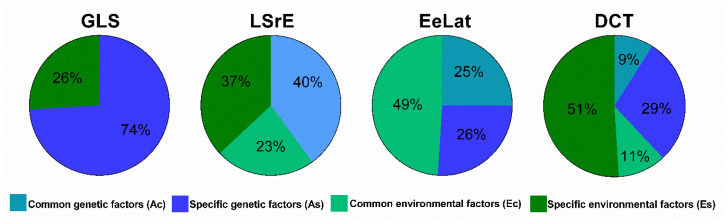
Schematic illustration of genetic and environmental influence on LV systolic and diastolic function using Systolo-Diastolic Model. Blue represents genetic influence, green environmental influence, the lighter shadow of each color represents specific effect, while the darker shadow the common effect. The sensitive left ventricular systolic parameter of global longitudinal strain (GLS) is mainly affected by specific genetic determinants (light blue) independent of diastolic parameters: LSrE (early diastolic strain rate), EeLat (lateral mitral annulus early diastolic tissue velocity), DCT (deceleration time of E wave). Although diastolic parameters are affected mainly by environment (all green shadows), LSrE and EeLat showed moderate genetic influence as well, proving a common effect only (dark blue) in the case of LSrE. All investigated diastolic parameters had common environmental influence (dark green).

In the third Systolo-Diastolic Model, investigating the major systolic and diastolic measures together, we found that GLS shares neither common genetic nor common environmental determination with diastolic parameters (Table 3). According to these results, GLS has a strong genetic background; however, both other components of myocardial deformation and diastolic function were determined independently from GLS in our population of middle-aged Caucasian twins (Figure 1).

The detailed model fit results showing the model that fitted the best to our data is presented in Supplementary Tables S1–S3 for the corresponding *Systolic*, *Diastolic*, and *Systolo-Diastolic Models*.

## 4. Discussion

We demonstrated mathematically that genetics have substantial impact on systolic LV function; however, none of the LV systolic parameters have any common genetic background, indicating that each systolic parameter has separate genetic determinants. Consequently, LV global longitudinal strain is independently inherited from circumferential strain, indicating that the strong inheritance (74%) of different myocardial layers is independent of each other. Diastolic function is defined mainly by environmental factors. Genetics have only modest influence on left ventricular diastolic function. The modest genetic influence (40%) on the accurate and sensitive LV early diastolic strain rate is mainly of common genetic background. However, the commonly used diastolic parameter of EeLat is affected 51% by genetics, which is equally shared between common and specific genetic determinants. Analyzing the co-heritability of LV systolic and diastolic parameters, we found that systolic and diastolic LV parameters have no common genetic or environmental determinants, suggesting that LV systolic and diastolic functional alterations may develop separately from each other.

The LV myocardium is composed of a continuous sheet of myofibers that are arranged in a multi-layered, helical manner. The corresponding deformation directions measured by speckle-tracking echocardiography have a strong, independent genetic and environmental determination, indicated by the fact that we found no common genetic and environmental roots for strain parameters. Regardless of the inherited or acquired nature of the etiology, myocardial damage usually starts with the most sensitive inner LV subendocardial fibers affecting longitudinal shortening; however, impairment of mid-wall muscle fibers contraction occurs at a later stage, influencing circumferential shortening [15]. Coronary artery disease, immune and metabolic factors, and exposure to toxins and pathogens are among the most common agents leading to myocardial damage, bearing its own genetic and environmental determining component. Nevertheless, genetic predisposition to environmental factor-induced myocardial impairment is another issue to consider. Genetic susceptibility to environmental damage, like cardiotoxicity from specific drugs, has long been investigated and still represents a challenging task, as early cardiotoxicity may be silent, yet its prompt diagnosis is important for patients with early structural heart changes but no signs or symptoms of heart failure [16,17].

We have shown that myocardial deformation resulting from the contraction of each of the aforementioned three layers has a strong, independent genetic determination. The diagnostic and prognostic value of longitudinal and circumferential strain in different cardiac abnormalities varies. GLS is a sensitive parameter for assessing LV function in the subclinical state of cardiovascular diseases when the conventional LV EF is still in normal range [18]. Our results suggest that independent genetic and environmental pathways contribute to each deformation direction. Moreover, the weak genetic background of LV diastolic parameters points at a higher susceptibility to environmental effects (i.e., comorbidities) of diastolic function. Similar to our results, the Belgium twin study and Korean twin and family study found low heritability of LV diastolic function [19,20]. This, along with an independent determination from systolic parameters, may also represent a potential background for such pathological states where the development of systolic and diastolic dysfunction is detached. Thus, assessing complex interactions of multiple quantitative traits may offer a better understanding of the development of various cardiac diseases, including heart failure with preserved ejection fraction, where systolic and diastolic dysfunction are often developing disconnected from each other.

The genetic component of LV systolic function using strain parameters was assessed in the Hypertension Genetic Epidemiology Network (HyperGEN) Study. In this large population- and family-based epidemiologic study, they found that longitudinal myocardial function is heritable, even after adjusting for several clinical factors associated with these indices [8]. Cardiac structure and function are important intermediate phenotypes that mediate the transition from risk factors (such as hypertension, obesity, and diabetes) to heart failure. Abnormalities in strain parameters have been shown to be associated with adverse outcomes [3]. The EchoGEN consortium of investigators published the first large-scale GWAS of cardiac structure and function in five community-based cohorts of European descent and identified only one locus (6q22), which achieved genome-wide significance, was successfully replicated, but only explained < 1% of the trait variance [21].

Several limitations of classical twin studies must be considered. A number of assumptions are made in structural equation modelling. It is assumed that MZ and DZ twin pairs share their environments to the same extent. Furthermore, it is assumed that twins are not different from the general population in terms of the examined traits and that the gene–environment interactions are minimal for these traits. Our results are drawn based on middle aged, relatively healthy Caucasian twins at low–moderate cardiovascular risk. Further studies, including twins treated for heart failure with preserved or reduced EF, might give a better insight into the inheritance and co-heritability of different LV functional alterations and their development. Our sample size was relatively modest; studies with larger sample sizes, including representation of multiple ethnicities, are warranted. Furthermore, zygosity in our twin cohort was determined based on validated questionnaires [22].

## 5. Conclusions

We demonstrated mathematically high heritability of LV systolic strain parameters with independent inheritance of each other. This substantial genetic determination of LV systolic biomechanics may help to preserve global LV systolic function for a longer period in the case of different environmental effects (for example, hypertension). The independent genetic determination of GLS, GCS, and basal and apical rotation might be one explanation of their separate deterioration in some diseases. However, LV diastolic function is much more sensitive to environmental changes, and diastolic dysfunction might be expressed independent of systolic dysfunction. Next-generation sequencing with whole exome and whole genome sequencing, as well as advances in epigenomics and analysis of gene–environment interactions will hopefully continue to improve our understanding of the genetics of cardiac structure and function.

## Figures and Tables

**Table 1:** Proportion of common and specific genetic and environmental factors contributing to heritability of systolic measurements.

Variable	GLS	GCS	Basal Rotation	Apical Rotation
**Common genetic and environmental factors**				
Genetic factors (A_C_)	0%	0%	0%	0%
Environmental factors (E_C_)	0%	0%	0%	0%
**Specific genetic and environmental factors**				
Genetic factors (A_S_)	76%	77%	57%	74%
Environmental factors (E_S_)	24%	23%	43%	36%
**Overall contribution of genetic and environmental factors**				
Genetic factors (A)	76%	77%	57%	74%
Environmental factors (E)	24%	23%	43%	36%

GLS: global longitudinal strain; GCS: global circumferential strain; Ac: common genetic factors; Cc: common environmental factors; Ec: common unique environmental factors; As: specific genetic factors; Cs: specific common environmental factors; Es: specific unique environmental factors.

**Table 2:** Proportion of common and specific genetic and environmental factors contributing to heritability of diastolic measurements.

Variable	LSrE	EeLat	DCT
**Common genetic and environmental factors**			
Genetic factors (A_C_)	40%	25%	9%
Environmental factors (E_C_)	23%	49%	11%
**Specific genetic and environmental factors**			
Genetic factors (A_S_)	0%	26%	29%
Environmental factors (E_S_)	37%	0%	51%
**Overall contribution of genetic and environmental factors**			
Genetic factors (A)	40%	51%	38%
Environmental factors (E)	60%	49%	62%

LSrE: early diastolic strain rate; EeLat: lateral mitral annulus early diastolic tissue velocity; DCT: deceleration time of E wave; Ac: common genetic factors; Cc: common environmental factors; Ec: common unique environmental factors; As: specific genetic factors; Cs: specific common environmental factors; Es: specific unique environmental factors.

**Table 3:** Proportion of common and specific genetic and environmental factors contributing to heritability of systolic and diastolic measurements.

Variable	GLS	LSrE	EeLat	DCT
**Common genetic and environmental factors**				
Genetic factors (A_C_)	0%	40%	25%	9%
Environmental factors (E_C_)	0%	23%	49%	11%
**Specific genetic and environmental factors**				
Genetic factors (A_S_)	74%	0%	26%	29%
Environmental factors (E_S_)	26%	37%	0%	51%
**Overall contribution of genetic and environmental factors**				
Genetic factors (A)	74%	40%	51%	38%
Environmental factors (E)	26%	60%	49%	62%

GLS: global longitudinal strain; LSrE: early diastolic strain rate; EeLat: lateral mitral annulus early diastolic tissue velocity; DCT: deceleration time of E wave; Ac: common genetic factors; Cc: common environmental factors; Ec: common unique environmental factors; As: specific genetic factors; Cs: specific common environmental factors; Es: specific unique environmental factors.

## Data Availability

The data presented in this study are available on request from the corresponding author.

## Supplementary Materials

The following are available online at https://www.mdpi.com/article/10.3390/medicina57090935/s1, Table S1: Detailed model information regarding multi-trait classical twin models of systolic measurements, Table S2: Detailed model information regarding multi-trait classical twin models of diastolic measurements, Table S3: Detailed model information regarding multi-trait classical twin models of systolic and diastolic measurements.
