# Supplementary material for: Left Ventricular Systolic Function Has Strong Independent Genetic Background from Diastolic Function: A Classical Twin Study
Source: Medicina (Kaunas). 2021 Sep 5;57(9):935. doi: 10.3390/medicina57090935 (PMC8470958; doi:10.3390/medicina57090935)
Supplement: Supplementary file 1 [file medicina-57-00935-s001.zip › medicina-1305031-supplementary.pdf]

Supplementary Table S1. Detailed model information regarding multi-trait classical twin models of systolic measurements.

Model number	Model name	Estimated parameters	Model *-2LL*	Model *df*	AIC	Difference to Full model *-2LL*	Difference to Full model *-df*	Difference to Full model *p*
1	Cholesky ACE	38	1866.23	674	518.23			
2	Cholesky AE	28	1866.74	684	498.74	0.50	10	1.00
3	Independent pathway ACE	32	1866.62	680	506.62	0.38	6	1.00
4	Independent pathway A_cE_c-$A_sC_sE_s$	28	1868.05	684	500.05	1.81	10	1.00
5	Independent pathway A_cE_c-A_sE_s	24	1868.05	688	492.05	1.81	14	1.00
6	Independent pathway A_c-A_sE_s	20	1874.45	692	490.45	8.21	18	0.98
7	Univariate	20	1876.00	692	492.00	9.76	18	0.94

Detailed results of calculated multi-trait structure equation models. -2LL: minus 2 log-likelihood value; AIC: Akaike information criteriondf: degrees of freedom. Ac: Common genetic factors; Cc: Common environmental factors; Ec: Common unique environmental factors; As: Specific genetic factors; Cs: Specific common environmental factors; Es: Specific unique environmental factors.

Supplementary Table S2. Detailed model information regarding multi-trait classical twin models of diastolic measurements

Model number	Model name	Estimated parameters	Model *-2LL*	Model *df*	AIC	Difference to Full model *-2LL*	Difference to Full model *-df*	Difference to Full model *p*
1	Cholesky ACE	24	1231.17	492	247.17	-	-	-
2	Cholesky AE	18	1231.44	498	235.44	0.27	6	1.00
3	Independent pathway ACE	24	1240.03	492	256.03	8.86	0	-
4	Independent pathway A_cE_c-$A_sC_sE_s$	21	1241.36	495	251.36	10.19	3	0.02
5	Independent pathway A_cE_c-A_sE_s	18	1241.367	498	245.36	10.19	6	0.12
6	Independent pathway A_cE_c-A_sE_s without A_c-LSrE	17	1241.36	499	243.36	10.19	7	0.18
7	Independent pathway A_c-A_sE_s	15	1278.05	501	276.05	46.873	9	$4.14*10^{-7}$
8	Univariate	15	1345.26	501	343.26	114.09	9	$2.15*10^{-20}$

Detailed results of calculated multi-trait structure equation models. -2LL: minus 2 log-likelihood value; AIC: Akaike information criteriondf: degrees of freedom. Ac: Common genetic factors; Cc: Common environmental factors; Ec: Common unique environmental factors; As: Specific genetic factors; Cs: Specific common environmental factors; Es: Specific unique environmental factors.

Supplementary Table S3. Detailed model information regarding multi-trait classical twin models of systolic and diastolic measurements

Model number	Model name	Estimated parameters	Model *-2LL*	Model *df*	AIC	Difference to Full model *-2LL*	Difference to Full model *-df*	Difference to Full model *p*
1	Cholesky ACE	38	1675.17	650	375.17			
2	Cholesky AE	28	1675.72	660	355.72	0.55	10	1.00
3	Independent pathway ACE	32	1687.60	656	375.60	12.43	6	0.05
4	Independent pathway A_cE_c-$A_sC_sE_s$	28	1689.98	660	369.98	14.80	10	0.14
5	Independent pathway A_cE_c-A_sE_s	24	1689.98	664	361.98	14.80	14	0.39
6	Independent pathway A_cE_c-A_sE_s without A_cE_c-GLS	22	1696.48	666	364.48	21.31	16	0.17
7	Independent pathway A_cE_c-A_sE_s without A_cE_c-GLS and A_s-LSrE	21	1696.48	667	362.48	21.31	17	0.21
8	Independent pathway A_c-A_sE_s	20	1726.45	668	390.45	51.28	18	$4.82*10^{-5}$
9	Univariate	20	1800.39	668	464.39	125.21	18	$4.32*10^{-18}$

Detailed results of calculated multi-trait structure equation models. -2LL: minus 2 log-likelihood value; AIC: Akaike information criteriondf: degrees of freedom. Ac: Common genetic factors; Cc: Common environmental factors; Ec: Common unique environmental factors; As: Specific genetic factors; Cs: Specific common environmental factors; Es: Specific unique environmental factors.
